# Real World Evidence: A Quantitative and Qualitative Glance at Participant Feedback from a Free-Response Survey Investigating Experiences of a Structured Exercise Intervention for Men with Prostate Cancer

**DOI:** 10.1155/2017/3507124

**Published:** 2017-07-03

**Authors:** L. Fox, F. Cahill, C. Burgess, N. Peat, S. Rudman, J. Kinsella, D. Cahill, G. George, A. Santaolalla, M. Van Hemelrijck

**Affiliations:** ^1^Cancer Epidemiology Group, Division of Cancer Studies, King's College London, London, UK; ^2^Physiotherapy, Guy's Hospital, Guy's and St Thomas' NHS Foundation Trust, London, UK; ^3^Medical Oncology, Guy's Hospital, Guy's and St Thomas' NHS Foundation Trust, London, UK; ^4^Royal Marsden, London, UK

## Abstract

**Aim:**

To explore patient experiences of a structured exercise intervention for men with prostate cancer (PCa).

**Sample:**

41 men with either localised or advanced PCa who had been referred for a structured exercise programme by their physician and then subsequently consented to a telephone survey.

**Method:**

Participants underwent a 10-week supervised exercise programme within a large cancer centre hospital consisting of 8 sessions. They then completed a short multiple choice telephone survey, elaborating on their responses where appropriate. Views expressed by participants were analysed using an affinity diagram and common themes were identified.

**Results:**

Feedback from our telephone surveys was consistently positive and suggests that the structured exercise intervention provides exercise confidence, motivation to exercise, and social support and promotes positive health behaviour change in the context of exercise. Individual differences arose amongst participants in their perceived utility of the intervention, with 73.3% expressing a preference for structured exercise classes and 19.5% expressing a preference for exercising independently.

**Conclusion:**

Design of a structured exercise intervention for patients with PCa should embrace the positive aspects outlined here but consider patients' individual differences. Ongoing feedback from patients should be utilised alongside traditional study designs to inform intervention design in this area.

## 1. Introduction

In the UK, prostate cancer (PCa) accounts for a quarter of all new male cancer diagnoses (46,690 in 2014) [1]. Individuals undergoing treatment for PCa can experience a multitude of physical and psychological issues that, coupled with the high incidence rate of PCa, form a high burden of disease. Moreover, PCa in men with metabolic conditions (i.e., with comorbidities of diabetes, obesity, or dyslipidaemia) is suggested to be linked with aggressive disease and death [2]. Not only is it thought that preexisting metabolic disorders increase the likelihood of a poor outcome for men who develop PCa, but androgen deprivation therapy (ADT, a standard treatment for advanced PCa) has also been linked to an increased risk of developing metabolic syndrome (MetS). When including only studies comparing men with PCa who were undergoing ADT to other PCa populations who were not undergoing ADT, our meta-analysis on risk of MetS after ADT for PCa [3] found a relative risk of 1.75 (95% CI: 1.27–2.41) for men undergoing ADT.

In recent years these observations have driven significant interest in physical exercise as an inexpensive and holistic adjuvant therapy that may help to improve physical and psychosocial outcomes [4] and survival time [5, 6]. There is evidence that engaging significantly with exercise can improve body composition, incontinence, muscular strength, cardiorespiratory fitness, and cancer-specific fatigue and quality of life (QoL) [7–10]. The salutary effects of exercise on depression are well documented [11]. Furthermore, exercise might provide a valuable antidote to feelings of helplessness, emasculation, and a lack of help-seeking behaviour that tends to be typical of men [12]. In addition, several studies have demonstrated that engagement with exercise following a PCa diagnosis can increase survival time [5, 6, 13]. For instance, a recent study using the Health Professionals Follow-up Study developed and applied a lifestyle score for prevention of lethal PCa [14]. Points were given in relation to smoking behaviour, BMI, physical activity, and diet. It was found that adhering to a healthy lifestyle, defined by not smoking, normal body weight, high physical activity, and a healthy diet, may lower risk of lethal PCa. Indeed, early in vitro evidence has suggested that serum taken from individuals who have been partaking in acute exercise may have an inhibitory effect on the growth of PCa cells [15]. Specific biological mechanisms underlying the apparent effect of exercise on prognosis are yet to be delineated and are currently under investigation [16, 17].

Thus, efforts are now underway to provide men with PCa with access to structured exercise classes as an adjunct to their usual care. Currently, most studies on exercise for PCa patients have small sample sizes, focus on a specific treatment of PCa, or have used self-report measures such as QoL questionnaires [18] or an objective quantitative outcome such as PCa survival [19–22]. It is recommended for the development of a complex intervention to take into account emergent insights about its implementation amongst the target population [23]. We therefore aimed to explore feedback from PCa patients (with both localised and advanced disease and undergoing various treatments) engaged with an ongoing pilot study of such structured exercise classes, with a view to report on the experiences of these men.

Previous qualitative research on men with PCa has provided evidence for physiological and psychosocial benefits of participating in a supervised exercise programme, although to our knowledge there is just one published qualitative study available that has focused on the experiences of an exercise intervention specifically amongst men with PCa. Using semistructured interviews, Cormie et al. [24] thematically analysed the interview responses of 12 men with PCa, following >3 months' involvement with a twice-weekly supervised aerobic and resistance exercise programme. Their analysis defined a number of psychosocial and physiological benefits to engagement with the programme, which they grouped into 3 overarching categories: health-related benefits, support from the exercise physiologists, and social support.

Cormie et al.'s study highlighted the importance of qualitative approaches to this area by presenting a number of insights that would not naturally translate into quantitative measurements. This influenced our approach; however the aim of the study we present here is not so much to collect evidence of the benefits of a supervised exercise intervention, but to gain further understanding of the best approach to maximising engagement with (and adherence to) exercise in general amongst this population, whether that be via a supervised exercise programme or independent exercise. A multivariate analysis by Courneya et al. [25] suggested that predictors of adherence to an exercise intervention for women with breast cancer may vary according to whether the exercise is supervised or unsupervised. They concluded that “predictors of adherence may differ for supervised and unsupervised exercise, moreover, predicting adherence to supervised exercise may be particularly difficult in well-controlled efficacy trials.” In the spirit of their latter point, the telephone surveys reported on here are an exploratory attempt to determine how much heterogeneity there may be in patients' attitudes to supervised/nonsupervised exercise and what factors may be driving this variance.

## 2. Methods

### 2.1. Study Population

**Figure 1:**
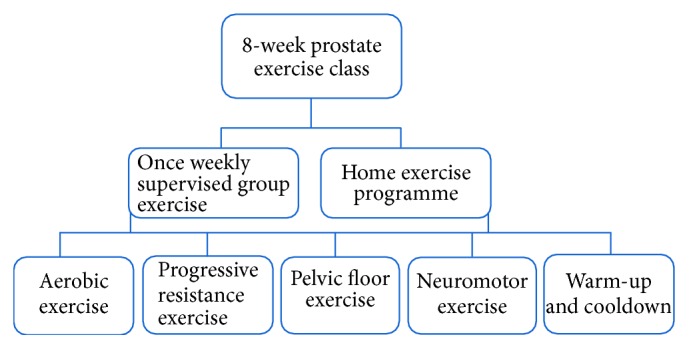
The prostate exercise class structure.

We included men with either localised or advanced PCa who were recommended by their physicians to engage with a physiotherapy-led structured exercise programme as a complement to their standard of care (Figure 1). Between November 2014 and September 2015, 76 men with PCa were referred to the physiotherapy team at Guy's Hospital (London, UK) for the structured exercise programme. Of these, 46 men completed the exercise programme, 10 dropped out before completing the programme, 10 declined to participate from the start (of whom three underwent initial assessment and then did not attend the classes), and one man died after agreeing to take part.

### 2.2. Exercise Intervention

Patients were invited to attend an exercise programme once a week for eight sessions over a 10-week period, allowing for the patient to miss up to two sessions as part of a pragmatic programme design. The rolling exercise programme was attended by 8–12 patients in each session and was modelled from existing published cancer exercise and UK physical activity recommendations [27, 28].

The intensity, load, and frequency of the exercise training were dependent on the physical function of the patient and also the current cancer treatment phase they were in (see Table 1). The exercise component consisted of a once weekly, 60-minute session, supervised by physiotherapists. The programme started with a warm-up, 5-10-minute standard flexibility regime to prevent injury. Combined circuit, of aerobic and progressive resistive exercise, was carried out for 40 minutes. Each circuit consisted of 3-minute exercise and 1-minute rest to allow for recovery and movement between each station. Neuromotor exercises (i.e., balance or coordination) and additional tailored exercises (i.e., pelvic floor muscle exercises) were prescribed as per individual need. Aerobic exercises were included aiming to maximise improvements in cardiorespiratory fitness and comprised of cross trainer, treadmill, arm cycle, and static cycling. The workload was defined by the heart rate reserve (HRR) using the Karvonen formula [29]. The low-to-moderate intensity (LMI) patient trained at 40–60% of HRR and the moderate-to-high intensity (MHI) patient trained at 60–80% of HRR. Patients were also encouraged to exercises between moderate to vigorous intensity using the Borg rating of perceived exertion scale [30]. Resistive exercises targeting large muscle groups consisted of leg press, leg extension, cable based multigym, dumbbells (ranging from 1 kg to 10 kg), and body weight (sit to stand, backward lunge, heel raises, and wall press). Resistive workload was defined by the one-repetition maximum (1-RM) measurement. LMI resistance exercises started in the first week at 40% of 1-RM and gradually increased to 60% of 1-RM in week 8, whereas MHI resistance exercises started at 60% of 1-RM gradually increasing to 70% of 1-RM by week 8. Each exercise was performed at 2-3 sets of 8–12 repetitions. The session ends with a cooldown, of 10-minute standard flexibility and relaxation regimen (standing or sitting). All exercises and exertion were scored and self-recorded by each patient.

In addition, three individualized sessions were provided at week 0 (prior to starting the exercise class), at session 4 (mid programme), and at session 8 (end of programme). These sessions were aimed at setting/reviewing training aims (i.e., workload, intensity, and frequency), applying behavioural motivational counselling techniques to enhance self-efficacy and overcome possible exercise barriers, and informing the patient of available services or resources on completion of the exercise programme.

Patients were encouraged (but not monitored) to complete two additional moderate intensity 30-minute exercise sessions by week 4 and three by week 8 as part of a home walking exercise programme. They were also given a pamphlet containing instructions on how to perform resistance exercises that they had been doing in the supervised sessions. The combination of our supervised exercise session and home-based programme aimed to encourage patients in meeting the recommendations of 150 minutes of physical activity for cancer survivors [27].

### 2.3. Surveys

Of the 76 men referred for the cancer exercise programme as of November 2016, 51 consented to involvement in our ongoing pilot study. Each of these men was contacted via phone in order to be interviewed as part of an audit process; all of them were contacted within the space of 2 weeks in November 2016, meaning that the time since completion of the exercise programme differed between participants within a range of approximately 6 months. 41 men were successfully contacted and all of these 41 men were willing to respond to a short telephone survey. None declined to take part in the survey. Men who agreed underwent a short interview about their experience of the exercise programme. Multiple choice questions that were put to the respondents are detailed in Table 3. Participants were also given the opportunity to elaborate on reasons for their responses, in an open-ended format that we hoped would offer some useful insight with regard to future refinement of our exercise intervention design.

As the participant feedback described here was obtained originally for audit purposes, the conversations that took place during the telephone survey were written down by researcher whilst conducting the interview. The information was thus not recorded and transcribed, but whilst we acknowledge that this limits the robustness of the findings presented here, we still obtained a valuable mixture of quantitative and qualitative data. The data proved to be informative with regard to our ongoing efforts to design an exercise intervention for men with prostate cancer. Participants provided responses to predetermined multiple choice questions, and notes were taken by the interviewing researcher that summarised any extra points made by each participant in the ensuing informal conversation. The data produced by this process enabled us to quantify to some extent the heterogeneity of responses to specific questions that we wanted to ask about the delivery of our exercise intervention, whilst leaving participants free to describe to us their first-hand experiences, which were of the utmost value in helping us to refine our intervention design and formulate further research questions. The notes produced by the researchers were analysed using an affinity diagram by another researcher (LF), and these themes were assessed and validated by a third researcher (CB).

An affinity diagram approach is a basic qualitative analysis method commonly used to inform management and planning [31]. In this process the source data (in this case, all of the notes taken by the interviewers) are divided into discrete units: for example, “participant describes a fun, social atmosphere at the classes” or “participant said the classes gave him the kick start he needed to get into routine exercise.” Units that are logically related are then grouped together, establishing any consistent themes. A theme was considered consistent if it was alluded to more than five times by participants. We were then able to examine our themes side by side with our quantified data from the multiple choice questions and draw some conclusions about how the exercise intervention could be refined or what research questions we should be asking going forward.

### 2.4. Ethics

Approval for a process evaluation/audit was obtained through the hospital's research and development department (project number: 47351).

## 3. Results

A description of the final sample of PCa patients that responded to our telephone survey is shown in Table 2. Responses to our multiple choice questions are shown in Table 3. Our affinity diagram produced from the notes that were taken on feedback offered by participants produced 5 common themes:* physiotherapy guidance*,* structured classes being a motivator*,* behaviour change*,* social support*, and* individual differences. *

### 3.1. Physiotherapy Guidance

The input of the physiotherapists was the most frequently identified valuable aspect of the structured classes. Indeed, the ability of the physiotherapists to give the participants exercise* confidence*, in the context of the physical and mental debilitation that can accompany PCa, was something frequently alluded to by interviewees. One participant said that the exercise class “feels like a safe environment” and another that the physiotherapists “cater to his care and needs.” Good-natured relationships with the physiotherapy team were reported frequently, and it was clear that the style of engagement of the physio team was a primary reason that the exercise classes were received so positively on the whole.

### 3.2. Structured Classes as a Motivator

It was apparent from our interviews that when it comes to engaging with physical exercise, the structured and tailored nature of the exercise classes acted as a strong motivating force. Of the 23 participants that told us that they felt* less* motivated when exercising independently, the responses of the vast majority highlighted two clearly dominant themes: the social environment that is provided with the structured classes and the potency of structured, timetabled appointments to provide an antidote to motivational inertia. Patients' descriptions of the motivational force of the group setting attributed this to both peer support and friendly intragroup competition.

### 3.3. Behaviour Change

Positive behavioural changes (defined in the context of the transtheoretical model of behaviour change [32]) were recorded by the physiotherapists in many patients participating in the structured exercise classes. These observations are consistent with a large number of responses from our interviews that were along the lines of “I wouldn't have engaged without the* kick start* that the structured classes gave me.” One participant, for example, said that he was initially sceptical, but the structured classes quickly changed his mind and now he continues to engage with the exercises he learnt in the class. Another participant said that the structured classes were a “massive boost” to his engagement with exercise, as he very quickly observed and felt the benefits. Another participant recalled being “taken aback” at how challenging the exercise programme was but went on to note that this was a positive experience. There were also a number of participants who admitted that, without the ongoing engagement with the structured classes, they would have been likely to quickly disengage with any initial efforts at routine physical exercise.

### 3.4. Social Support

One of the aspects of the structured exercise classes that participants were most enthused about was the social value of the classes. Our interviews drew a picture of an environment that is good-humoured and enjoyable. One interviewee referred to fellow class participants as his “friends.” Some also told of the value of having someone to relate to (i.e., other men with PCa). One of the interviewees drew this into sharp focus: he had been told by his doctor that he needed to engage with exercise but had been too depressed to adhere to this advice alone; he went on to say that the class enabled him to talk with another patient who had been through the classes already and had lost weight and got fit. The interviewee described this encounter as a strong antidote to the inertia he was experiencing as a result of his depression.

### 3.5. Individual Differences

Both participant feedback and the multiple choice responses indicated that individual personality traits seemed to be strongly influential in determining how useful a structured exercise intervention would be for that person. Most of the participants (73.3%) seemed to prefer the structured classes. This figure is comparable to that of Gjerset et al. (2011), which looked at exercise interests and preferences in cancer survivors [33]. However, almost 20% of participants said that they would prefer to exercise independently, with many of these participants giving substantive reasons relating to individual personality traits or lifestyle, for instance, having the ability to motivate oneself or integrating exercise into their recreational activities.

Some of the individual differences we note are a likely result of our offered exercise intervention not meeting individual preferences. Although our programme design took a proactive approach when determining the intensity, load, and frequency of the exercise training, the chosen mode was a predetermined circuit set of exercises within a supervised group setting. Research has cited that exercise interests and intervention preferences can vary in cancer populations with the most reported preferred types of exercises including walking, resistance exercises, activities of recreation, and exercises of a moderate intensity [34, 35]. Furthermore, cancer survivors have a preference on how exercise programmes are delivered. Unlike our study, a 2002 study of cancer survivors by Jones and Corneya reported that 40% of the people studied expressed a preference for exercising within their own home [34].

We noted that some of the participants who were already previously engaged with physical exercise still found the classes to be a valuable component of their care, for example, as a way to facilitate reengagement with physical exercise following radical surgery. We also observed that participants' responses to question 6, “If your doctor recommended exercise to you to improve treatment outcome, and you were not offered exercise classes within the hospital, would you have been more/less likely to exercise (or the same either way)?,” were fairly evenly distributed, highlighting differences between participants in the way in which they might respond to exercise recommendations from their doctor.

## 4. Discussion

Informal feedback from open-ended surveys with the PCa patients in our ongoing pilot study has provided some preliminary insights into how best to devise a structured exercise intervention for all men with PCa. Overall across our sample we observed that participant feedback regarding the structured exercise classes was strongly positive. Our interview responses suggest that there may be multiple benefits to patients of running such a service, namely, the provision of motivation to exercise, exercise guidance, and social support, and it is possibly a catalyst for positive health behaviour change relating to exercise. Some heterogeneity in interviewees' responses suggested that patients' individual differences should be taken into account when designing interventions of this type, indicating that personal disposition, personal circumstances, and exercise history may be useful candidates for predicting of the likelihood of adherence to a particular exercise programme, or receptiveness to a particular way of presenting the intervention to them (e.g., via the nursing team rather than the patient's consultant).

Bourke et al. have assessed the effects of exercise on cancer-specific quality of life and adverse events in PCa trials [10]. They concluded from 16 randomised controlled trials (RCTs) involving 1,574 men with PCa that exercise interventions are efficacious in improving cancer-specific QoL, fatigue, and exercise capacity in men with PCa. The evidence to date comes mainly from men on ADT. However, there is a lack of data on effectiveness and cost-effectiveness of PCa exercise interventions when integrated into healthcare services [10]. There are still some ongoing trials [36, 37] but common limitations of these are small sample sizes as well as a focus on only one specific treatment for PCa. The Individualized Diet and Exercise Adherence Pilot Trial (IDEA-P) includes 40 patients with PCa undergoing ADT. Sheffield University is setting up an exercise training intervention as a novel primary therapy for men with localised prostate cancer only: PANTERA (Prostate cAncer Novel ThERApy) trial [37]. To our knowledge very little information is available today about the patient experience and preferences for these exercise interventions. In addition to the existing qualitative and quantitative measurements, the insights obtained from these interviews will improve our understanding of how best to encourage men with PCa throughout their cancer pathway to undertake exercise. Below we describe the different themes observed in our responses in the context of our quantitative multiple choice responses and existing research.

Individual differences were pronounced in the feedback we received. Lifestyle factors and personality traits appeared to influence preference. It could be speculated that men more used to exercising independently may feel that exercising independently gives them more control over their engagement, whereas the reverse may be true for men who were not previously exercising regularly: they may feel as though the structured classes empower, rather than inhibit, them. The latter was certainly true for some of the participants in this study. Preferences for individually tailored interventions have been expressed in previous studies [38, 39]. For instance, a Canadian study set up focus groups with 27 men on ADT and identified that men were interested in a mobile application to reduce sedentary behaviour designed to be easy to use, have an alerting function to interrupt sitting, have the ability to track and monitor physical activity levels, to be tailored to the individual, and involve social support [39]. There is also research suggesting that predictors of adherence may vary according to whether exercise programmes are supervised or unsupervised [4, 25]. These findings are somewhat consistent with our interpretation.

Individuals also expressed differences in how they respond to different styles of advice, expressed clearly by the mixed responses that we received to question 6. This was reflected in various comments from participants. According to some, the advice of a consultant would be effective enough to get them to engage with exercise behaviour, whereas others expressed a level of endearment to, and trust of, the nursing team that would make them more likely to adhere to their instructions than their consultant. It is possible that this distribution of responses to question 6 represents both (a) variance in the quality of the participants' relationships with their particular doctor and (b) variance in dispositional attitudes towards the medical profession amongst participants. Two studies have found that around two-thirds of their cancer patient samples were interested in participating in exercise [40, 41], yet one of these, conducted in Canada, also found that 70% had received no exercise counselling throughout their cancer care [40]. It has been suggested that this is likely due to a lack of knowledge amongst clinicians about exercise for prostate cancer and that contact with clinical exercise physiologists could benefit patients [4]. An Australasian survey of 119 oncology nurses reported that two of the primary barriers to physical exercise promotion were lack of time and lack of adequate support structures [42]. Considering this in terms of our own experience, our cancer-specific exercise intervention currently receives patients via interdepartmental referrals from urology to cancer physiotherapy, and it may be that further integration of the physiotherapy team into the urology clinic/multidisciplinary team could facilitate patient engagement with exercise programmes, by promoting awareness amongst clinicians and providing a proximal person for patients to talk to specifically about exercise, before any further referral is made. Further integration of this type may help to accommodate for individual differences in the way prostate cancer patients respond to exercise advice.

It was clear that some of these men with PCa felt a lack of confidence in their ability to exercise effectively. As well as motivation, the physiotherapy team were able to provide personalised clinical supervision and guidance to patients that let them engage in exercise knowing they were not putting their health at risk. To the best of our knowledge, there is a lack of published research on cancer-related fear avoidance behaviour. Some of our interviewees, however, described what appeared to be fear avoidance behaviours relating to overexertion in light of their cancer diagnosis. We can speculate that the negation of this fear via the support of the physiotherapists is one of the reasons why around three-quarters (73.3%) of our participants expressed a preference for attending structured classes, despite the inconvenience of the weekly travel to the hospital. In other chronic disease settings, it has already been shown that fear avoidance beliefs may be useful in identifying patients at risk of psychosocial problems as well as their pain intensity and physical impairment [43]. Hence, challenging cancer-related fear avoidance behaviour that could be detrimental to physical and mental wellbeing may be beneficial to PCa patients, and so there may be scope for research in this area. It will be interesting to see the results from a Norwegian trial on early rehabilitation of patients with breast, colorectal, prostate, or testicular cancer [44]. This RCT will provide empirical evidence of whether an individually administered stress management programme can decrease stress and maintain or enhance patients' physical activity level, quality of life, and psychological wellbeing.

It was clear from the participant feedback that the structured classes provided additional motivation to exercise. The structured nature of appointments, on a certain day and time, made patients feel obliged to attend, which requires less willpower that attempting to engage with exercise independently. In addition, the physiotherapists are skilled at driving patients to push themselves harder physically then they may do otherwise. Around two-thirds (65.8%) of our participants were pushed harder than they had expected to be and yet still maintained their attendance. Furthermore, there is likely a social aspect to motivation: some patients were making friends in the class, which could arguably make attending the classes feel more attractive for some. The opportunity for social interaction within group exercise has been described previously by clinical populations as a motivator to attend [45, 46]. Conversely, it is possible that more introverted individuals may not find the group setting attractive. The latter was not a view expressed by any of our interviewees but may be worth considering. A 2014 systematic review found that there was a dearth of research into social influences on exercise adherence in cancer patients and recommended this as a future avenue of inquiry [47]. Our interviews would appear to support their recommendations.

Our interview responses suggested that, for some men, attending these classes may provide a crucial social support network. Friendships were being formed via attendance at the classes and patients may find particular solace in their social interactions with fellow patients in the class that they may not be able to find elsewhere. This observation is consistent with previous research involving women with breast cancer, which suggested that the action-oriented format of an exercise class may provide a preferable way to acknowledge cancer in a social context, as opposed to simply talking about it [48]. It is likely that this sentiment could be more strongly expressed in men. Given previous research suggesting that exercise engagement can be used to facilitate engagement with counselling via partnerships with physiotherapists and counsellors [49], an exercise programme could be a valuable tool in addressing the psychosocial issues facing men with PCa, both within the class and via counselling.

This study was limited in the sense that, due to constraints on the methodology, the results reported are more descriptive than analytical. However, insight from patients is a necessary component of the development of a complex intervention [23]. Combined with quantitative and qualitative data of existing and ongoing studies, this work can generate data on how best to support PCa patients in terms of exercise empowerment. Our population consisted predominantly of Caucasian men, so future research will have to ensure a better representation of different ethnic groups. It is however the case that black men are consistently underrepresented in clinical trials, and this is an ongoing challenge [50]. It is also possible that our responses suffer from selection bias as those willing to participate may also have a more positive attitude towards exercise interventions.

## 5. Conclusion

This work on patients' views and experiences has generated UK data on how best to support PCa patients in terms of exercise empowerment. The spectrum of insights obtained highlighted some components that can be considered when designing exercise interventions for men with PCa, with individual tailoring being the most commonly suggested need. Future research into understanding how best to encourage men with PCa throughout their cancer pathway to undertake exercise should combine emerging insights from immediate feedback with more general qualitative observations and quantitative measurements. Research questions addressing exercise adherence in the PCa population may do well to acknowledge the value of service and facilities provision such as that outlined here, in particular the social aspects of such provision and the role it can play in exercise confidence/fear avoidance behaviour, motivation, and health behaviour change. Our observations suggest that those who are looking to implement this type of service provision should acknowledge these factors, whilst appreciating that preferred approaches to exercise engagement can vary largely between individuals.

## Figures and Tables

**Table 1:** Exercise intensities of low-to-moderate intensity (LMI) and moderate-to-high (MHI) intensity endurance and resistance prescription related to cancer treatment and physical level.

Intensity level	Patient description	HRR	BORG (RPE) 0–10	1-RM
LMI	(i) On active cancer treatment (ii) <8 weeks posttreatment (iii) ^*∗*^Reduced physical level	40–60%	3-4	40–60%

MHI	(i) On established hormone therapy (ii) 8 weeks after completion of active treatment (iii) ^*∗*^Expected physical level	60–80%	4–6	60–70%

HRR, heart rate reserve; BORG (0–10), modified rating of perceived exertion scale; 1-RM, one-repetition maximum. ^*∗*^Physical level was determined on completion of a 6-minute time walk test [26] and a functional assessment before commencing the exercise classes. Where training intensity seemed too high or too low, the HRR, BORG, or 1-RM was reassessed.

**Table 2:** Description of the final sample of PCa patients on the exercise programme who were contactable and agreed to a phone interview. All values are baseline (preexercise programme).

	Total	Localised disease	Advanced disease
	41	20	21
Age			
Minimum	43	43	52
Maximum	80	70	80
* Mean (SD)*	*63.63 (9.24)*	*59.2 (8.38)*	*67.9 (8.15)*
Initial PSA			
Minimum	0.4	0.4	4.1
Maximum	3196	67.45	3196
* Mean (SD)*	*138.15 (509.74)*	*10.7 (14.03)*	*265.6 (706.37)*
Initial Gleason score			
6	10 (24.39%)	9 (45%)	1 (4.76%)
7	18 (43.9%)	10 (50%)	8 (38.1%)
8	5 (12.2%)	1 (5%)	4 (19.05%)
9	7 (17.07%)	0	7 (33.33%)
10	1 (2.44%)	0	1 (4.76%)
Current/most recent treatment			
Active surveillance	2 (4.88%)	2 (10%)	0
ADT	10 (24.39%)	0	10 (47.62%)
Brachytherapy	1 (2.44%)	0	1 (4.76%)
Chemotherapy	5 (12.2%)	0	5 (23.81%)
Radiotherapy	3 (7.32%)	1 (5%)	2 (9.52%)
RALRP	16 (39.02%)	13 (65%)	3 (14.29%)
Awaiting RALRP	3 (7.32%)	3 (15%)	0
TURP	1 (2.44%)	1 (5%)	0
Ethnicity			
White/Caucasian	35 (85.4%)	16 (80%)	19 (90.5%)
Black/Afro-Caribbean	3 (7.3%)	2 (10%)	1 (4.8%)
Asian	1 (2.4%)	0	1 (4.8%)
Other	2 (4.9%)	2 (10%)	0
Marital status			
Married	28 (68.3%)	15 (75%)	13 (61.9%)
Divorced/separated	6 (14.6%)	1 (5%)	5 (23.8%)
Widowed	3 (7.3%)	1 (5%)	2 (9.5%)
Never married	4 (9.8%)	3 (15%)	1 (4.8%)
Current living circumstances			
Alone	6 (14.6%)	4 (20%)	2 (9.5%)
With partner	32 (78%)	16 (80%)	16 (76.2%)
With other family	2 (4.9%)	0	2 (9.5%)
Other	1 (2.4%)	0	1 (4.8%)
Current work circumstances			
Full-time	10 (24.4%)	8 (40%)	2 (9.5%)
Part-time	5 (12.2%)	2 (10%)	3 (14.3%)
Retired	24 (58.5%)	9 (45%)	15 (71.4%)
Unemployed	1 (2.4%)	1 (5%)	0
* (Missing data)*	*1 (2.4%)*	*0*	*1 (4.8%)*
>150 mins of moderate exercise per week			
Yes	35 (85.4%)	14 (70%)	21 (100%)
No	6 (14.6%)	6 (30%)	0
Approx. sedentary minutes per week			
Minimum	90	90	240
Maximum	4800	4200	4800
* Mean (SD)*	*1459 (1190)*	*1175 (1220)*	*1729 (1123)*

**Table 3:** Responses to multiple choice questions.

*Q1. Would you take part again, if offered hospital-based exercise classes again?*		
Yes	39	95.2%
No	2	4.8%

*Q2. Do you prefer …*		
Exercising independently, outside of the hospital	8	19.5%
Exercising using hospital facilities, with minimal support	2	4.8%
Structured exercise classes within the hospital	30	73.3%
No preference	1	2.4%

*Q3. When doing independent exercise outside of the hospital, rather than supervised exercise, do you feel more motived to exercise, or less motivated?*		
More motivated	15	36.6%
Less motivated	23	56.1%
The same	3	7.3%

*Q4. Was your prior perception of how hard you would be pushed on the exercise programme accurate?*		
Yes	14	34.2%
No	27	65.8%

*Q5. If we had given you an advice leaflet on exercise, and you were not offered exercise classes within the hospital, would you have been …*		
More likely to exercise	4	9.8%
Less likely to exercise	25	61.0%
The same either way	12	29.2%

*Q6. If your doctor had recommended exercise to you to improve treatment outcome, and you were not offered exercise classes within the hospital, would you have been …*		
More likely to exercise	10	24.4%
Less likely to exercise	14	34.2%
The same either way	17	41.4%

*Q7. If your doctor had NOT recommended exercise to you to improve treatment outcome, but you were offered exercise classes within the hospital within the nursing team, would you have been …*		
More likely to exercise	31	75.6%
Less likely to exercise	2	4.8%
The same either way	8	19.6%
